# Mr. Atkinson, on the Use of the Seton

**Published:** 1806-09

**Authors:** James Atkinson

**Affiliations:** York


### *To the Editors of the Medical and Phyſical Journal.*

Gentlemen,

Having found considerable advantage from the use of the seton after operations on the eyes, especially after couching; I hope the recommendation of it to the Faculty in those cases, may not be deemed intruding upon the sheets of your valuable Journal.

If my experience in many instances had not clearly convinced convinced me of the propriety and benefit of the Seton, I should not have presumed to bring it forward, particularly as it was a very ancient plan of cure in many other disorders.

The inflammations which take place after operations upon the eyes, are well known to produce, on some occasions, abscesses, suffusions, contractions of the iris, &c. &c. These do not invariably give way to local or general bleeding, or indeed to any other attempts. Loss of sight will sometimes succeed, in defiance of the means of the most skilful operators.

To prevent such accidents, or to relieve them when threatening, is the intention of the seton. The result of my experience speaks highly in its favour.

In all operations upon this organ, where there may be reason to apprehend subsequent injury, it has been my practice to introduce a seton in the neck, either at the time of the operation, or after a few days. The seton appears to me, for this intention, more effectual than any other endeavour which I have used.

In some cases it was not employed, until a certain degree of pain, of inflammation, of eccymosis, or of swelling in the eye-lids and membranes actually existed.

These complaints, in general, have given way more or less speedily to the use of the seton; and it has been a satisfactory and most excellent resource to me, upon many occasions.

Later experience has induced me to insert the silk on the very day of operation; and also to adopt it in other cases, to resist the attack of inflammation, &c. upon the eyes.

Surgeons of former times were more versed than we are, in the use of the seton: it is not therefore, the novelty of the practice which you will permit me to recommend, but the proper application.

It may be set on the back of the neck as usual; or if deemed more eligible, very near, or within the roots of the hair. And however objectionable the operation may prove to young persons, yet, in cases of need, it will, I hope, be found particularly useful.

Gentlemen, will you excuse a digression from the subject. I wish much to urge the expediency of inoculating for the vaccine, in parts less exposed than the arm.

Any surface of the skin, it is to be presumed, will answer. I have many years ago inoculated in the leg successfully cessfully for the small-pox, and of late equally so, for the vaccine, just above the knee, on the inside of the thigh.

It will avoid disfiguring a part, which the fashion of dress, at present, requires to be exposed.

Experience may determine the best situation for its insertion. Possibly, such may be found, where the lymphatics take their course most clear of glands.

I do not apprehend, that any person has already advised the adoption of this mode of practice.

Is there any strong objection to it? Perhaps some gentlemen may think it worth the trial.

I am, &c.

JAMES ATKINSON.

*York, July* 8, 1806.

